# Crystal structure of 2-ethyl-3-(4-fluoro­phenyl­sulfon­yl)-5,7-dimethyl-1-benzo­furan

**DOI:** 10.1107/S1600536814019436

**Published:** 2014-09-24

**Authors:** Hong Dae Choi, Uk Lee

**Affiliations:** a Department of Chemistry, Dongeui University, San 24 Kaya-dong, Busanjin-gu, Busan 614-714, Republic of Korea; b Department of Chemistry, Pukyong National University, 599-1 Daeyeon 3-dong, Nam-gu, Busan 608-737, Republic of Korea

**Keywords:** crystal structure, benzo­furan, 4-fluoro­phen­yl, sulfon­yl, hydrogen bonds, π–π inter­actions.

## Abstract

In the title compound, C_18_H_17_FO_3_S, the dihedral angle between the plane of the benzo­furan ring [r.m.s. deviation = 0.006 (1) Å] and that of the 4-fluoro­phenyl ring [r.m.s. deviation = 0.004 (1) Å] is 82.45 (4)°. In the crystal, mol­ecules are linked *via* three different pairs of C—H⋯O hydrogen bonds, forming chains along [001] and enclosing two *R* ^2^_2_(10) and one *R* ^2^_2_(12) ring motifs. The chains are further linked by π–π inter­actions [inter-centroid distance = 3.566 (1) Å] between the furan rings of inversion-related mol­ecules, forming a two-dimensional network lying parallel to (100).

## Abstract 2

In the title compound, the dihedral angle between the plane of the benzo­furan ring and the 4-fluoro­phenyl ring is 82.45 (4)°. In the crystal, mol­ecules are linked *via* three different pairs of C—H⋯O hydrogen bonds, forming chains along [001] and enclosing two 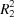
(10) and one 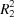
(12) ring motifs.

## Chemical Context   

Substituted benzo­furans show important pharmacological properties such as anti­bacterial and anti­fungal, anti­tumour and anti­viral, and anti­microbial activities (Aslam *et al.* 2009 ▶; Galal *et al.*, 2009 ▶; Khan *et al.*, 2005 ▶), and are potential inhibit­ors of β-amyloid aggregation (Howlett *et al.*, 1999 ▶; Ono *et al.*, 2002 ▶). These benzo­furan compounds occur in a great number of natural products (Akgul & Anil, 2003 ▶; Soekamto *et al.*, 2003 ▶). As a part of our ongoing project concerning 2-alkyl-3-(phenyl­sulfon­yl)-5,7-dimethyl-1-benzo­furan derivatives, we report herein on the synthesis and crystal structure of the title compound.
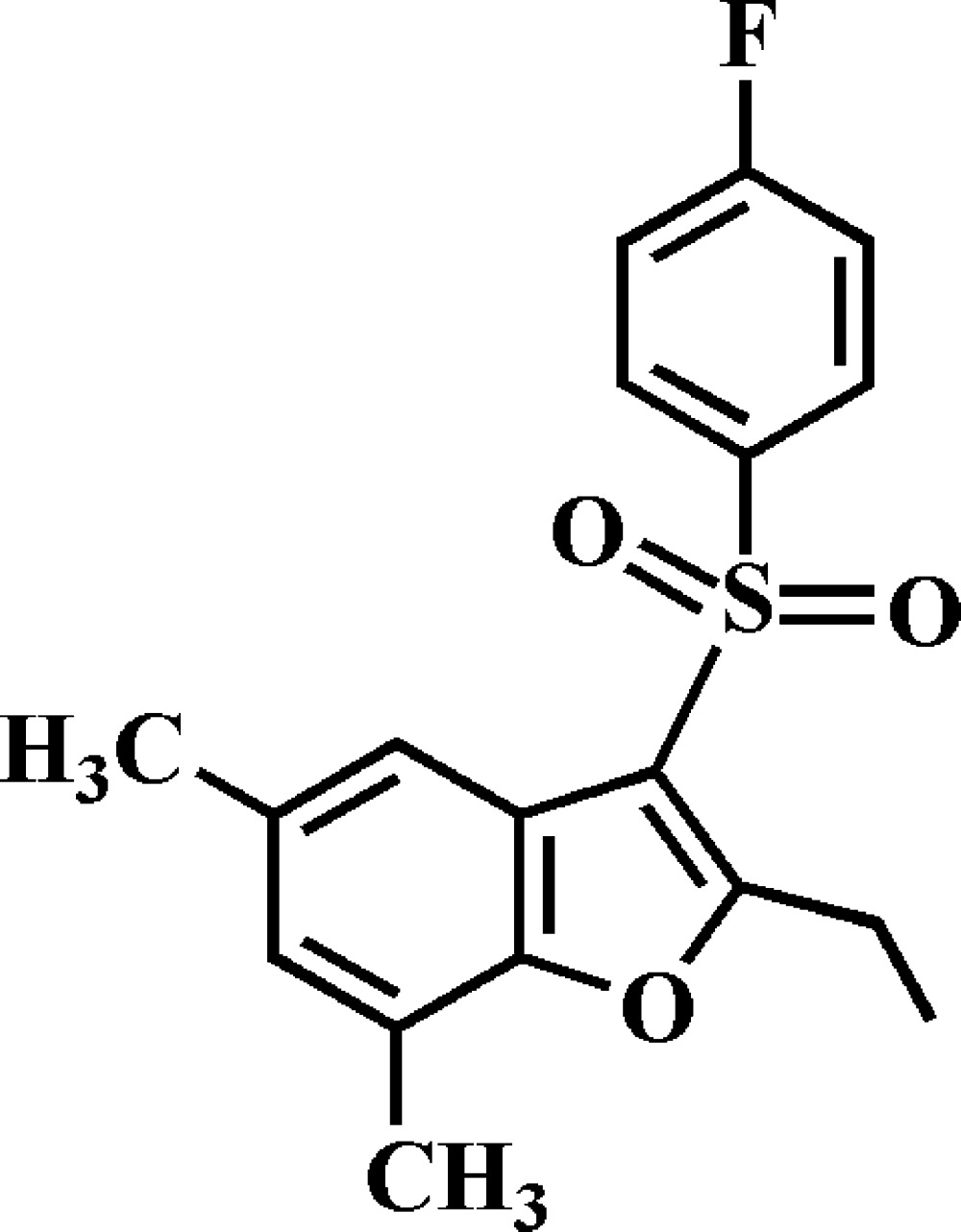


## Structural commentary   

**Figure 1:**
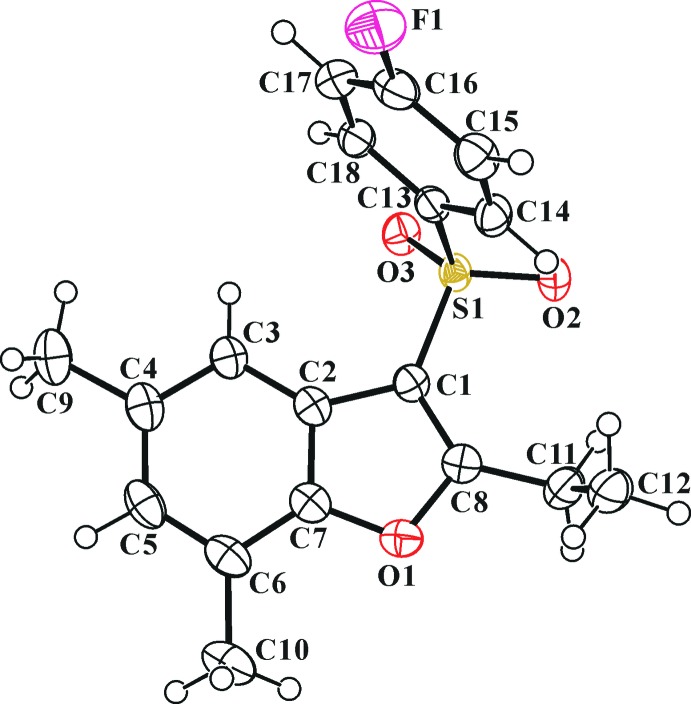
The mol­ecular structure of the title mol­ecule, with atom labelling. Displacement ellipsoids are drawn at the 50% probability level.

In the title mol­ecule, Fig. 1 ▶, the benzo­furan unit (O1/C1–C8) is essentially planar, with a mean deviation of 0.006 (1) Å from the mean plane defined by the nine constituent atoms. The 4-fluoro­phenyl ring (C13–C18) is inclined to the benzo­furan ring by 82.45 (4)°.

## Supra­molecular features   

**Figure 2:**
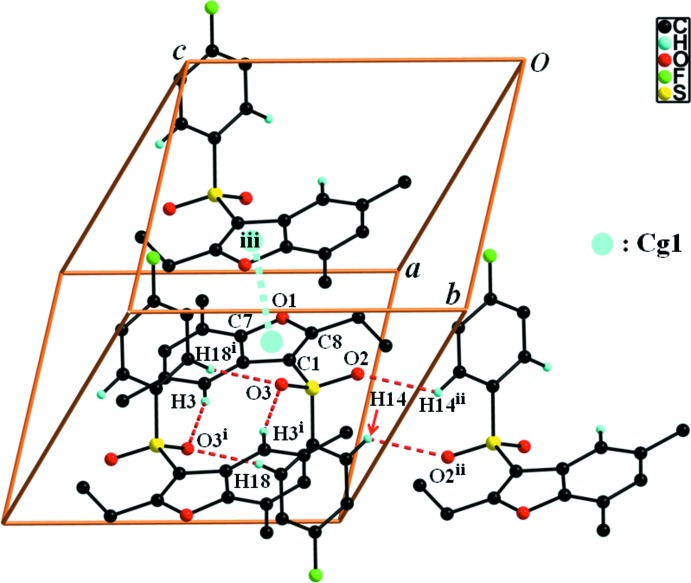
A view of the C—H⋯O and π–π inter­actions (dotted lines) in the crystal structure of the title compound [see Table 1 ▶ for details; H atoms not involved in hydrogen bonding have been omitted for clarity; symmetry codes: (i) −*x* + 1, −*y* + 2, −*z* + 1; (ii) −*x* + 1, −*y* + 2, −*z*; (iii) −*x* + 1, −*y* + 1, −*z* + 1].

In the crystal, mol­ecules are linked *via* three different pairs of C—H⋯O hydrogen bonds, forming chains along [001] and enclosing two 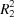
(10) and one 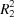
(12) ring motifs (Fig. 2 ▶ and Table 1 ▶). The chains are further linked by π–π inter­actions between the furan rings of inversion-related mol­ecules, forming a two-dimensional network lying parallel to (100) [illustrated in Fig. 2 ▶; *Cg*1⋯*Cg*1^i^ = 3.566 (1), inter­planar distance = 3.553 (1); slippage = 0.305 Å; *Cg*1 is the centroid of the C1/C2/C7/O1/C8 furan ring; symmetry code: (i) −*x* + 1, −*y* + 1, −*z* + 1].

## Database survey   

A search of the Cambridge Structural Database (Version 5.35, last update May 2014; Allen, 2002 ▶) for 3-(phenyl­sulfon­yl)benzo­furan gave 65 hits. Six of these involve 5,7-dimethyl-3-(phenyl­sulfon­yl)benzo­furan derivatives. They include the 2-methyl derivative of the title compound, 2-methyl-3-(4-fluoro­phenyl­sulfon­yl)-5,7-dimethyl-1-benzo­furan (Choi *et al.*, 2010 ▶). In these six compounds, the dihedral angle between the phenyl­sulfonyl ring and the benzo­furan ring varies from *ca*. 72.68° in the 2-methyl derivative mentioned above, to 87.61° in 2-methyl-3-(2-fluoro­phenyl­sulfon­yl)-5,7-dimethyl-1-benzo­furan (Choi *et al.*, 2014 ▶). The same angle in the title compound is 82.45 (4)°.

## Synthesis and crystallization   

The starting material 2-ethyl-3-(4-fluoro­phenyl­sulfan­yl)-5,7-dimethyl-1-benzo­furan was prepared by a literature method (Choi *et al.* 1999 ▶). 3-Chloro­per­oxy­benzoic acid (77%, 448 mg, 2.0 mmol) was added in small portions to a stirred solution of 2-ethyl-3-(4-fluoro­phenyl­sulfan­yl)-5,7-dimethyl-1-benzo­furan (270 mg, 0.9 mmol) in di­chloro­methane (35 ml) at 273 K. After being stirred at room temperature for 8h, the mixture was washed with saturated sodium bicarbonate solution (2 × 15 ml) and the organic layer was separated, dried over magnesium sulfate, filtered and concentrated at reduced pressure. The residue was purified by column chromatography (hexa­ne–ethyl acetate, 4:1 *v*/*v*) to afford the title compound as a colourless solid [yield 61% (236 mg); m.p. 416–417 K; *R*_f_ = 0.63 (hexa­ne–ethyl acetate, 4:1 *v*/*v*)]. Single crystals suitable for X-ray diffraction were prepared by slow evaporation of a solution of the title compound (21 mg) in acetone (15 ml) at room temperature. ^1^H NMR (δ p.p.m., CDCl_3_, 400 Hz): 7.99–8.04 (*m*, 2H), 7.47 (*s*, 1H), 7.14–7.19 (*m*, 2H), 6.93 (*s*, 1H), 3.22 (*q*, *J* = 7.52 Hz, 2H), 2.43 (*s*, 3H), 2.41 (*s*, 3H), 1.36 (*t*, *J* = 7.54 Hz, 3H).

## Refinement   

Crystal data, data collection and structure refinement details are summarized in Table 2 ▶. All H atoms were positioned geometrically and refined as riding atoms: C—H = 0.95 Å for aryl, 0.99 Å for methyl­ene and 0.98 Å for methyl H atoms, respectively, with *U*_iso_(H) = 1.5*U*_eq_(C) for methyl H atoms and = 1.2*U*_eq_(C) for other H atoms.

## Supplementary Material

Crystal structure: contains datablock(s) I. DOI: 10.1107/S1600536814019436/zp2015sup1.cif

Structure factors: contains datablock(s) I. DOI: 10.1107/S1600536814019436/zp2015Isup2.hkl

Supporting information file. DOI: 10.1107/S1600536814019436/zp2015Isup3.cml

CCDC reference: 1021511

Additional supporting information:  crystallographic information; 3D view; checkCIF report

## Figures and Tables

**Table 1:** Hydrogen-bond geometry (Å, °)

*D*—H⋯*A*	*D*—H	H⋯*A*	*D*⋯*A*	*D*—H⋯*A*
C3—H3⋯O3^i^	0.95	2.55	3.4804 (18)	167
C14—H14⋯O2^ii^	0.95	2.49	3.1211 (17)	124
C18—H18⋯O3^i^	0.95	2.36	3.2742 (17)	160

Symmetry codes: (i) 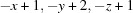; (ii) 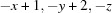.

**Table 2:** Experimental details

Crystal data	
Chemical formula	C_18_H_17_FO_3_S
*M*_r_	332.38
Crystal system, space group	Triclinic, *P* 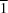
Temperature (K)	173
*a*, *b*, *c* (Å)	8.8756 (2), 9.3917 (2), 11.0284 (2)
α, β, γ (°)	65.735 (1), 80.735 (1), 71.145 (1)
*V* (Å^3^)	792.68 (3)
*Z*	2
Radiation type	Mo *K*α
μ (mm^−1^)	0.23
Crystal size (mm)	0.39 × 0.33 × 0.30

Data collection	
Diffractometer	Bruker *SMART* APEXII CCD
Absorption correction	Multi-scan (*SADABS*; Bruker, 2009 ▶)
*T*_min_, *T*_max_	0.918, 0.936
No. of measured, independent and observed [*I* > 2σ(*I*)] reflections	14813, 3934, 3489
*R*_int_	0.025
(sin θ/λ)_max_ (Å^−1^)	0.668

Refinement	
*R*[*F* ^2^ > 2σ(*F* ^2^)], *wR*(*F* ^2^), *S*	0.037, 0.108, 1.07
No. of reflections	3934
No. of parameters	211
H-atom treatment	H-atom parameters constrained
Δρ_max_, Δρ_min_ (e Å^−3^)	0.29, −0.44

Computer programs: *APEX2* and *SAINT* (Bruker, 2009 ▶), *SHELXS97* and *SHELXL2014* (Sheldrick, 2008 ▶), *ORTEP-3 for Windows* (Farrugia, 2012 ▶), *DIAMOND* (Brandenburg, 1998 ▶) and *PLATON* (Spek, 2009 ▶).

## supplementary crystallographic information

### Crystal data

**Table:**

C_18_H_17_FO_3_S	*Z* = 2
*M**_r_* = 332.38	*F*(000) = 348
Triclinic, *P*1	*D*_x_ = 1.393 Mg m^−^^3^
Hall symbol: -P 1	Melting point = 417–416 K
*a* = 8.8756 (2) Å	Mo *K*α radiation, λ = 0.71073 Å
*b* = 9.3917 (2) Å	Cell parameters from 6147 reflections
*c* = 11.0284 (2) Å	θ = 2.4–28.2°
α = 65.735 (1)°	µ = 0.23 mm^−^^1^
β = 80.735 (1)°	*T* = 173 K
γ = 71.145 (1)°	Block, colourless
*V* = 792.68 (3) Å^3^	0.39 × 0.33 × 0.30 mm

### Data collection

**Table:**

Bruker SMART APEXII CCD diffractometer	3934 independent reflections
Radiation source: rotating anode	3489 reflections with *I* > 2σ(*I*)
Graphite multilayer monochromator	*R*_int_ = 0.025
Detector resolution: 10.0 pixels mm^-1^	θ_max_ = 28.4°, θ_min_ = 2.0°
φ and ω scans	*h* = −11→11
Absorption correction: multi-scan (*SADABS*; Bruker, 2009)	*k* = −12→12
*T*_min_ = 0.918, *T*_max_ = 0.936	*l* = −14→14
14813 measured reflections	

### Refinement

**Table:**

Refinement on *F*^2^	Primary atom site location: structure-invariant direct methods
Least-squares matrix: full	Secondary atom site location: difference Fourier map
*R*[*F*^2^ > 2σ(*F*^2^)] = 0.037	Hydrogen site location: difference Fourier map
*wR*(*F*^2^) = 0.108	H-atom parameters constrained
*S* = 1.07	*w* = 1/[σ^2^(*F*_o_^2^) + (0.0587*P*)^2^ + 0.2229*P*] where *P* = (*F*_o_^2^ + 2*F*_c_^2^)/3
3934 reflections	(Δ/σ)_max_ < 0.001
211 parameters	Δρ_max_ = 0.29 e Å^−^^3^
0 restraints	Δρ_min_ = −0.44 e Å^−^^3^

### Special details

**Table:**

Geometry. All esds (except the esd in the dihedral angle between two l.s. planes) are estimated using the full covariance matrix. The cell esds are taken into account individually in the estimation of esds in distances, angles and torsion angles; correlations between esds in cell parameters are only used when they are defined by crystal symmetry. An approximate (isotropic) treatment of cell esds is used for estimating esds involving l.s. planes.
Refinement. Refinement of F^2^ against ALL reflections. The weighted R-factor wR and goodness of fit S are based on F^2^, conventional R-factors R are based on F, with F set to zero for negative F^2^. The threshold expression of F^2^ > 2sigma(F^2^) is used only for calculating R-factors(gt) etc. and is not relevant to the choice of reflections for refinement. R-factors based on F^2^ are statistically about twice as large as those based on F, and R- factors based on ALL data will be even larger.

### Fractional atomic coordinates and isotropic or equivalent isotropic displacement parameters (Å^2^)

**Table:**

	*x*	*y*	*z*	*U*_iso_*/*U*_eq_	
S1	0.47686 (4)	0.90767 (4)	0.29373 (3)	0.02612 (11)	
F1	0.95796 (13)	1.25062 (13)	0.04333 (11)	0.0519 (3)	
O1	0.69433 (12)	0.44317 (12)	0.38093 (10)	0.0329 (2)	
O2	0.37720 (12)	0.93598 (13)	0.19098 (10)	0.0339 (2)	
O3	0.40523 (12)	0.95254 (13)	0.40432 (10)	0.0331 (2)	
C1	0.58429 (16)	0.70342 (16)	0.35637 (13)	0.0268 (3)	
C2	0.68621 (15)	0.62616 (16)	0.46953 (13)	0.0274 (3)	
C3	0.72804 (16)	0.67484 (18)	0.55984 (13)	0.0300 (3)	
H3	0.6840	0.7834	0.5555	0.036*	
C4	0.83619 (17)	0.5600 (2)	0.65658 (14)	0.0343 (3)	
C5	0.89864 (17)	0.40020 (19)	0.66131 (15)	0.0374 (3)	
H5	0.9724	0.3241	0.7282	0.045*	
C6	0.85859 (17)	0.34717 (18)	0.57367 (15)	0.0352 (3)	
C7	0.75119 (16)	0.46619 (17)	0.47913 (14)	0.0304 (3)	
C8	0.59290 (16)	0.58923 (17)	0.30738 (14)	0.0296 (3)	
C9	0.8875 (2)	0.6073 (2)	0.75486 (16)	0.0472 (4)	
H9A	0.8299	0.7212	0.7398	0.071*	
H9B	1.0023	0.5946	0.7432	0.071*	
H9C	0.8636	0.5370	0.8456	0.071*	
C10	0.9270 (2)	0.17627 (19)	0.57832 (19)	0.0461 (4)	
H10A	0.8523	0.1503	0.5397	0.069*	
H10B	0.9449	0.0998	0.6710	0.069*	
H10C	1.0284	0.1673	0.5273	0.069*	
C11	0.52011 (18)	0.58876 (19)	0.19537 (15)	0.0364 (3)	
H11A	0.4181	0.6764	0.1747	0.044*	
H11B	0.4962	0.4836	0.2233	0.044*	
C12	0.6270 (2)	0.6136 (2)	0.06985 (16)	0.0425 (4)	
H12A	0.6419	0.7225	0.0361	0.064*	
H12B	0.5772	0.6031	0.0020	0.064*	
H12C	0.7307	0.5311	0.0907	0.064*	
C13	0.62269 (16)	1.01143 (16)	0.21923 (13)	0.0261 (3)	
C14	0.68862 (17)	1.01386 (17)	0.09555 (13)	0.0306 (3)	
H14	0.6562	0.9596	0.0527	0.037*	
C15	0.80202 (18)	1.09615 (19)	0.03520 (14)	0.0348 (3)	
H15	0.8485	1.0998	−0.0495	0.042*	
C16	0.84549 (18)	1.17233 (17)	0.10114 (15)	0.0349 (3)	
C17	0.7805 (2)	1.17300 (18)	0.22324 (15)	0.0363 (3)	
H17	0.8130	1.2283	0.2650	0.044*	
C18	0.66666 (17)	1.09115 (17)	0.28363 (14)	0.0307 (3)	
H18	0.6194	1.0895	0.3677	0.037*	

### Atomic displacement parameters (Å^2^)

**Table:**

	*U*^11^	*U*^22^	*U*^33^	*U*^12^	*U*^13^	*U*^23^
S1	0.02580 (17)	0.02980 (17)	0.02171 (17)	−0.00369 (12)	−0.00149 (12)	−0.01186 (13)
F1	0.0532 (6)	0.0496 (6)	0.0541 (6)	−0.0289 (5)	0.0052 (5)	−0.0128 (5)
O1	0.0315 (5)	0.0285 (5)	0.0380 (5)	−0.0076 (4)	0.0000 (4)	−0.0131 (4)
O2	0.0317 (5)	0.0415 (6)	0.0287 (5)	−0.0063 (4)	−0.0073 (4)	−0.0145 (4)
O3	0.0316 (5)	0.0399 (5)	0.0275 (5)	−0.0046 (4)	0.0029 (4)	−0.0185 (4)
C1	0.0262 (6)	0.0289 (6)	0.0241 (6)	−0.0070 (5)	0.0012 (5)	−0.0104 (5)
C2	0.0243 (6)	0.0301 (6)	0.0240 (6)	−0.0092 (5)	0.0027 (5)	−0.0069 (5)
C3	0.0280 (6)	0.0354 (7)	0.0243 (6)	−0.0101 (5)	0.0016 (5)	−0.0092 (5)
C4	0.0288 (7)	0.0445 (8)	0.0244 (6)	−0.0135 (6)	0.0014 (5)	−0.0067 (6)
C5	0.0288 (7)	0.0398 (8)	0.0293 (7)	−0.0094 (6)	−0.0023 (6)	0.0008 (6)
C6	0.0271 (7)	0.0299 (7)	0.0371 (8)	−0.0084 (5)	0.0021 (6)	−0.0028 (6)
C7	0.0265 (6)	0.0307 (7)	0.0303 (7)	−0.0100 (5)	0.0028 (5)	−0.0081 (5)
C8	0.0266 (6)	0.0313 (6)	0.0306 (7)	−0.0084 (5)	0.0018 (5)	−0.0124 (6)
C9	0.0453 (9)	0.0623 (11)	0.0312 (8)	−0.0132 (8)	−0.0081 (7)	−0.0144 (8)
C10	0.0366 (8)	0.0301 (7)	0.0568 (10)	−0.0065 (6)	−0.0028 (7)	−0.0042 (7)
C11	0.0357 (7)	0.0402 (8)	0.0403 (8)	−0.0100 (6)	−0.0021 (6)	−0.0226 (7)
C12	0.0564 (10)	0.0399 (8)	0.0350 (8)	−0.0134 (7)	−0.0004 (7)	−0.0187 (7)
C13	0.0284 (6)	0.0242 (6)	0.0225 (6)	−0.0029 (5)	−0.0030 (5)	−0.0086 (5)
C14	0.0339 (7)	0.0341 (7)	0.0252 (6)	−0.0077 (6)	−0.0014 (5)	−0.0139 (6)
C15	0.0358 (7)	0.0366 (7)	0.0279 (7)	−0.0081 (6)	0.0023 (6)	−0.0114 (6)
C16	0.0339 (7)	0.0274 (6)	0.0368 (8)	−0.0090 (6)	−0.0029 (6)	−0.0052 (6)
C17	0.0464 (8)	0.0290 (7)	0.0359 (8)	−0.0101 (6)	−0.0072 (6)	−0.0133 (6)
C18	0.0382 (7)	0.0281 (6)	0.0253 (6)	−0.0055 (5)	−0.0033 (5)	−0.0121 (5)

### Geometric parameters (Å, º)

**Table:**

S1—O2	1.4353 (10)	C9—H9B	0.9800
S1—O3	1.4383 (10)	C9—H9C	0.9800
S1—C1	1.7341 (14)	C10—H10A	0.9800
S1—C13	1.7648 (14)	C10—H10B	0.9800
F1—C16	1.3520 (17)	C10—H10C	0.9800
O1—C8	1.3684 (17)	C11—C12	1.525 (2)
O1—C7	1.3833 (18)	C11—H11A	0.9900
C1—C8	1.3630 (19)	C11—H11B	0.9900
C1—C2	1.4482 (18)	C12—H12A	0.9800
C2—C7	1.3892 (19)	C12—H12B	0.9800
C2—C3	1.3935 (19)	C12—H12C	0.9800
C3—C4	1.392 (2)	C13—C14	1.3891 (18)
C3—H3	0.9500	C13—C18	1.3901 (19)
C4—C5	1.403 (2)	C14—C15	1.385 (2)
C4—C9	1.505 (2)	C14—H14	0.9500
C5—C6	1.389 (2)	C15—C16	1.373 (2)
C5—H5	0.9500	C15—H15	0.9500
C6—C7	1.385 (2)	C16—C17	1.379 (2)
C6—C10	1.503 (2)	C17—C18	1.385 (2)
C8—C11	1.485 (2)	C17—H17	0.9500
C9—H9A	0.9800	C18—H18	0.9500
			
O2—S1—O3	119.08 (6)	C6—C10—H10A	109.5
O2—S1—C1	108.98 (6)	C6—C10—H10B	109.5
O3—S1—C1	108.06 (6)	H10A—C10—H10B	109.5
O2—S1—C13	108.05 (6)	C6—C10—H10C	109.5
O3—S1—C13	107.27 (6)	H10A—C10—H10C	109.5
C1—S1—C13	104.43 (6)	H10B—C10—H10C	109.5
C8—O1—C7	107.20 (11)	C8—C11—C12	113.00 (13)
C8—C1—C2	108.02 (12)	C8—C11—H11A	109.0
C8—C1—S1	127.10 (11)	C12—C11—H11A	109.0
C2—C1—S1	124.84 (10)	C8—C11—H11B	109.0
C7—C2—C3	119.68 (13)	C12—C11—H11B	109.0
C7—C2—C1	104.35 (12)	H11A—C11—H11B	107.8
C3—C2—C1	135.96 (13)	C11—C12—H12A	109.5
C4—C3—C2	118.14 (14)	C11—C12—H12B	109.5
C4—C3—H3	120.9	H12A—C12—H12B	109.5
C2—C3—H3	120.9	C11—C12—H12C	109.5
C3—C4—C5	119.77 (15)	H12A—C12—H12C	109.5
C3—C4—C9	120.17 (15)	H12B—C12—H12C	109.5
C5—C4—C9	120.05 (14)	C14—C13—C18	121.55 (13)
C6—C5—C4	123.62 (14)	C14—C13—S1	118.74 (10)
C6—C5—H5	118.2	C18—C13—S1	119.70 (10)
C4—C5—H5	118.2	C15—C14—C13	119.42 (13)
C7—C6—C5	114.32 (14)	C15—C14—H14	120.3
C7—C6—C10	122.25 (15)	C13—C14—H14	120.3
C5—C6—C10	123.43 (14)	C16—C15—C14	118.19 (13)
O1—C7—C6	125.00 (14)	C16—C15—H15	120.9
O1—C7—C2	110.53 (12)	C14—C15—H15	120.9
C6—C7—C2	124.47 (14)	F1—C16—C15	118.29 (14)
C1—C8—O1	109.89 (12)	F1—C16—C17	118.31 (14)
C1—C8—C11	135.05 (13)	C15—C16—C17	123.41 (14)
O1—C8—C11	115.06 (12)	C16—C17—C18	118.51 (13)
C4—C9—H9A	109.5	C16—C17—H17	120.7
C4—C9—H9B	109.5	C18—C17—H17	120.7
H9A—C9—H9B	109.5	C17—C18—C13	118.92 (13)
C4—C9—H9C	109.5	C17—C18—H18	120.5
H9A—C9—H9C	109.5	C13—C18—H18	120.5
H9B—C9—H9C	109.5		
			
O2—S1—C1—C8	8.30 (15)	C3—C2—C7—C6	−0.5 (2)
O3—S1—C1—C8	139.05 (12)	C1—C2—C7—C6	179.20 (13)
C13—S1—C1—C8	−106.97 (13)	C2—C1—C8—O1	−0.30 (15)
O2—S1—C1—C2	−173.96 (10)	S1—C1—C8—O1	177.75 (9)
O3—S1—C1—C2	−43.20 (13)	C2—C1—C8—C11	−179.77 (15)
C13—S1—C1—C2	70.78 (12)	S1—C1—C8—C11	−1.7 (2)
C8—C1—C2—C7	0.46 (14)	C7—O1—C8—C1	0.01 (15)
S1—C1—C2—C7	−177.64 (10)	C7—O1—C8—C11	179.60 (11)
C8—C1—C2—C3	−179.96 (15)	C1—C8—C11—C12	95.6 (2)
S1—C1—C2—C3	1.9 (2)	O1—C8—C11—C12	−83.88 (16)
C7—C2—C3—C4	0.70 (19)	O2—S1—C13—C14	−38.40 (12)
C1—C2—C3—C4	−178.83 (14)	O3—S1—C13—C14	−167.95 (10)
C2—C3—C4—C5	−0.4 (2)	C1—S1—C13—C14	77.52 (12)
C2—C3—C4—C9	178.89 (13)	O2—S1—C13—C18	140.33 (11)
C3—C4—C5—C6	−0.1 (2)	O3—S1—C13—C18	10.79 (13)
C9—C4—C5—C6	−179.43 (14)	C1—S1—C13—C18	−103.75 (12)
C4—C5—C6—C7	0.4 (2)	C18—C13—C14—C15	0.6 (2)
C4—C5—C6—C10	179.40 (14)	S1—C13—C14—C15	179.36 (11)
C8—O1—C7—C6	−179.37 (13)	C13—C14—C15—C16	0.2 (2)
C8—O1—C7—C2	0.30 (15)	C14—C15—C16—F1	179.00 (13)
C5—C6—C7—O1	179.55 (12)	C14—C15—C16—C17	−1.0 (2)
C10—C6—C7—O1	0.5 (2)	F1—C16—C17—C18	−179.19 (13)
C5—C6—C7—C2	−0.1 (2)	C15—C16—C17—C18	0.8 (2)
C10—C6—C7—C2	−179.13 (13)	C16—C17—C18—C13	0.1 (2)
C3—C2—C7—O1	179.87 (11)	C14—C13—C18—C17	−0.8 (2)
C1—C2—C7—O1	−0.47 (14)	S1—C13—C18—C17	−179.53 (11)

### Hydrogen-bond geometry (Å, º)

**Table:**

*D*—H···*A*	*D*—H	H···*A*	*D*···*A*	*D*—H···*A*
C3—H3···O3^i^	0.95	2.55	3.4804 (18)	167
C14—H14···O2^ii^	0.95	2.49	3.1211 (17)	124
C18—H18···O3^i^	0.95	2.36	3.2742 (17)	160

Symmetry codes: (i) −*x*+1, −*y*+2, −*z*+1; (ii) −*x*+1, −*y*+2, −*z*.
